# Mapping tobacco industry strategies in South East Asia for action planning and surveillance

**DOI:** 10.1136/tc.2006.017988

**Published:** 2008-01-18

**Authors:** F Stillman, M Hoang, R Linton, B Ritthiphakdee, W Trochim

**Affiliations:** 1 Institute for Global Tobacco Control, Johns Hopkins University, Baltimore, MD, USA; 2 Union Institute and University, Cincinnati, OH, USA; 3 South East Asia Tobacco Control Alliance, Bangkok, Thailand; 4 Cornell University, Ithaca, NY, USA

## Abstract

**Objective:**

To develop a comprehensive conceptual framework of tobacco industry tactics in four countries in South East Asia for the purpose of: (1) generating consensus on key areas of importance and feasibility for regional and cross country tobacco industry monitoring and surveillance; (2) developing measures to track and monitor the effects of the tobacco industry and to design counterstrategies; and (3) building capacity to improve tobacco control planning in the participating countries.

**Design:**

A structured conceptualisation methodology known as concept mapping was used. The process included brainstorming, sorting and rating of statements describing industry activities. Statistical analyses used multidimensional scaling and cluster analysis. Interpretation of the maps was participatory, using regional tobacco control researchers, practitioners, and policy makers during a face to face meeting.

**Participants:**

31 participants in this study come from the four countries represented in the project along with six people from the Johns Hopkins Blomberg School of Public Health.

**Conclusions:**

The map shows eight clusters of industry activities within the four countries. These were arranged into four general sectors: economics, politics, public relations and deception. For project design purposes, the map indicates areas of importance and feasibility for monitoring tobacco industry activities and serves as a basis for an initial discussion about action planning. Furthermore, the development of the map used a consensus building process across different stakeholders or stakeholder agencies and is critical when developing regional, cross border strategies for tracking and surveillance.

South East Asia has one of the highest annual per capita cigarette consumption growth rates (range of 2–8%) among the six World Health Organization regions.^1^ The smoking prevalence among males in many countries in South East Asia is over 50%. In addition, the region has very low smoking rates among females and a very large proportion of the population is under 18 years of age—very appealing target markets for the tobacco industry. Another attractive quality for the multinational tobacco companies is the relatively permissive legislative environment—with few countries in the region having developed or implemented tobacco control policies, such as restrictions on production, sales, and advertising of tobacco products. However, Thailand, one of the region’s leaders in tobacco control, has been the exception. Thailand has banned cigarette advertising, restricts free sampling of cigarettes, limits tobacco sponsorship and has put into place graphic health warnings. However, most countries in the region have more permissive tobacco control legislation. For example, Cambodia does not regulate advertising content or design, sponsorship by tobacco companies is allowed, there is no ban on sale to minors and free sampling of cigarettes or other tobacco products is allowed.

Restrictions on advertising are important, especially now as countries in South East Asia implement the ASEAN (Association of Southeast Asian Nations) Free Trade Agreement (AFTA) and come into compliance with the World Trade Organization’s (WTO) requirements. In the absence of strong tobacco control legislation and enforcement, trade liberalisation has been shown to have a large and significant impact on cigarette consumption in low income countries.^2^ An example of this is Thailand’s experience of a 10% increase in cigarette consumption following reduction of trade barriers that allowed US cigarettes into the country.^2^

The release of internal industry documents and the advent of the Framework Convention on Tobacco Control (FCTC) have, however, forced the industry to polish up its image, streamline its marketing strategies and affirm its role as a valuable contributor to the economy. Experience has shown that the monitoring of tobacco companies and exposing industry’s deceptions has been useful in enacting effective tobacco control policies.^3^ It is useful to have a high level strategic view of industry tactics so that tobacco control planners can better anticipate the tactics the industry might use and also identify effective counter-measures such as policy development, enforcement and monitoring.

This paper describes a planning process to assist four countries in South East Asia to develop a systematic approach to track and monitor tobacco industry activities locally and regionally. A similar project was undertaken to map tobacco industry strategies in the United States, but this has not been done for any other part of the world.^3^ This initiative was part of a larger initiative supported by the Rockefeller Foundation entitled “Trading Tobacco for Health (TTFH).” The Institute for Global Tobacco Control (IGTC) of the Johns Hopkins University Bloomberg School of Public Health worked with the regional partners to develop national and regional tobacco industry surveillance capacities and tracking tools. Concept mapping was used to develop a conceptual framework of tobacco industry activities as a basis for initial discussion about action planning. The process was also used to assess areas of tobacco control considered both important and feasible for tracking and surveillance.

## METHODS

A structured conceptualisation methodology known as concept mapping was used as the core methodology for the project. Concept mapping is a participatory, mixed methods approach that combines group process activities with multivariate statistics. The process can be used to help a group describe its ideas on any topic of interest and represent these ideas visually in the form of a map.^4^ ^5^ Participants brainstorm a large set of statements relevant to the topic of interest, individually sort these statements into piles of similar ones, rate each statement on one or more scales and interpret the maps that result from the data analyses. Multidimensional scaling and hierarchical cluster analysis are used to yield both statistical and graphic representations of the conceptual domains. Participants are led through a structured interpretation session designed to help them understand the graphic representation (map) and group participation is used to label the map in a substantively meaningful manner.

### Procedures

There are four distinct phases in the process: brainstorming, sorting and rating, data analysis and generation of the maps, and expert panel interpretation of the maps. In-depth explanations of the methods and procedures are described elsewhere.^6^ ^7^ The entire process for the South East Asia concept mapping was accomplished between August and December 2003. All analyses were conducted and maps produced using the Concept System computer software that was designed for this process.^8^

### Participants

The brainstorming phase had 35 participants while the sorting and rating phase had 31 participants. A convenience sample yielded participants who were selected because they were active members of the South East Asia Tobacco Control Alliance (SEATCA), had been engaged in training workshops of the SEATCA, had been involved in tobacco control research and/or programming and demonstrated understanding of tobacco industry strategies at work in their countries. The majority were from the four countries participating in the TTFH project—that is, Cambodia (n = 5), Malaysia (n = 8), Thailand (n = 7) and Vietnam (n = 5). In addition, six members of staff from the IGTC participated; other participants were (n = 4) from international agencies with direct knowledge of the region and the countries. Of all the participants, 11 were advocates, 6 were policy makers, and 18 were tobacco control researchers. The mean number of years involved in tobacco control work was 4.5 (ranging from 1 to 17). The brainstorming and the sorting and rating were facilitated in person in each of the countries in the study by one of the authors and were accomplished using paper and pencil or actual printed stacks of cards. The interpretation of the maps took place at the Regional Workshop on Monitoring and Surveillance to Advance Tobacco Control Policies, 15–17 December 2003, Bangkok, Thailand.

### Brainstorming

The list of tactics that the tobacco industry uses in South East Asia was generated using a brainstorming approach.^9^ ^10^ The brainstormed statements were generated in response to a specific prompt: “A specific activity that the tobacco industry uses to block tobacco control in South East Asia is......” Participants were encouraged to generate as many statements as possible and to write down their statements on a piece of paper. The statements were then shared with the group. Participants were not allowed to challenge or question the statements of others (although they could ask for clarifications). The intent was to generate as many industry activities as possible. The primary brainstorming occurred at an international tobacco control conference in Helsinki and included a diverse group of tobacco control and public health experts, including representatives of the four participating countries (Cambodia, Malaysia, Thailand and Vietnam). Additionally, brainstorming was also done with an international group of tobacco control experts via email. The statements were compiled, edited (removing duplicates) and synthesised by one of the authors into a final set of 86 statements. Table 1 lists all of the statements generated.

**Table 1:** South East Asia tobacco control statements

(1) Get pro-tobacco information into scientific literature		(44) Promote pro-tobacco candidates for government offices
(2) Promote tobacco control focus that is limited to youth issues and youth education programmes		(45) Exploit regional trade agreements
(3) Create youth antismoking campaigns		(46) Conduct meetings with ministries of finance and health to influence opinion on tobacco control issues
(4) Harass tobacco control advocates		(47) Argue that tobacco tax increases encourage smuggling
(5) Diversify investments to protect themselves economically (entering into food, alcohol and clothing businesses)		(48) Focus attention towards ineffective public policies and programmes (for example, youth restricted access, youth antismoking campaigns)
(6) Develop and fund environmental health agencies (for example, the Institute for Air Quality (IAQ), environmental front groups)		(49) Promote tobacco products through use of young, pretty girls
(7) Evade advertising bans through trans-border broadcasting		(50) Invest in traditional home production business of tobacco products—bidis, kreteks, etc
(8) Provide tobacco farmers with technical assistance (use of fertilisers, processing, etc)		(51) Target poor by images associating tobacco with success and freedom
(9) Create ineffective anti-smoking school programmes		(52) Undermine the FCTC process (for example, by providing governments with written suggested responses)
(10) Support “front groups” for the tobacco industry		(53) Push for weak health warning labels on tobacco products
(11) Provide government officials with contributions, gifts or special perks		(54) Pressure governments to develop joint ventures between locally owned companies and multinational tobacco companies
(12) Provide retailers with youth educational materials		(55) Provide money to government programmes and initiatives to gain political favour
(13) Publicise philanthropy contributions		(56) Avoid legislative interventions by promoting self regulation (for example, tobacco industry marketing standards)
(14) Evade taxes by basing taxation on weight vs number of sticks in pack		(57) Fund researchers to present at tobacco control conferences
(15) Co-opt scientists working in toxicology and environmental health and safety		(58) Circumvent ad bans through indirect promotions and sponsorships
(16) By providing funds for their education and training		(59) Circumvent ad bans through brand stretching
(17) Threaten to withdraw financial support from government programmes		(60) Sponsor sports and music concerts
(18) Lobby for passing weak tobacco control laws and restrictions		(61) Divert attention from health issues by focusing attention on economic issues
(19) Create doubt and confusion regarding the science of environmental tobacco smoke		(62) Infiltrate key research and educational institutions (such as WHO) by training their professional staff and consultants
(20) Facilitate tobacco smuggling as a way to counter tax increases		(63) Disregard regulations on ingredient disclosure
(21) Lobby ministries of tourism, industry and trade		(64) Develop allies with powerful élites
(22) Promote “Courtesy of Choice” and other accommodation programmes		(65) Engage in free sampling of tobacco products
(23) Create alliances in the private sector retailers, vendors, hospitality		(66) Use economic clout to buy media coverage
(24) Organise local conferences about indoor air quality to confuse science		(67) Conduct meetings with FCTC national delegations to influence opinion
(25) Avoid taxes by requesting “tax holiday” after capital investment		(68) Promote ventilation programmes instead of indoor smoking bans
(26) Establish friendly relationships with government officials, policy makers or tobacco control advocates		(69) Highlight philanthropic contributions (for example, medical missions and development programmes)
(27) Argue that tobacco production and sales reduce poverty		(70) Assert economic benefits of the tobacco industry to the country
(28) Co-opt youth organisations and school programmes to implement youth anti-smoking campaigns		(71) Write educational curriculum at prestigious institutions
(29) Pressure governments to privatise tobacco industry		(72) Purchase tobacco from offshore accounts
(30) Argue that increases in tobacco taxes will reduce government revenues		(73) Publicise corporate and social responsibility activities to enhance public
(31) Destroy industry documents		(74) Assert that tobacco taxes are regressive and anti-poor
(32) Assert that higher tobacco tax threatens job security and employment, especially for poor farmers		(75) Purchase medical research institutions
(33) Use university professors to lobby government officials		(76) Provide financial support to key institutions such as the International Monetary Fund and the World Health Organization
(34) Oppose increases in tobacco taxes		(77) Develop alliances with the hospitality industry
(35) Work against the ratification of the FCTC		(78) Fund reforestation campaigns to divert accusations of environmental damage
(36) Write weak tobacco control legislation for governments		(79) Target women through the use of western images of female empowerment
(37) Influence scientific discourse by infiltrating academic institutions		(80) Hire consultants to promote industry view on scientific issues
(38) Silence tobacco control news and information		(81) Write indoor air policies that are consistent with industry policies on accommodation and ventilation
(39) Falsely compliance with tobacco control rules and regulations		(82) Threaten local policy makers that they will lose in the elections if they do not support the industry
(40) Buy allegiance of future scientific experts by supporting their undergraduate and graduate school training		(83) Develop display and promotional materials at point of purchase
(41) Hire individuals to enter into tobacco control community and create fractions (disunite)		(84) Promote tobacco through free giveaways
(42) Misrepresent tobacco control issues to naive reporters		(85) Use capitol investments to bargain for relief from tobacco control measures
(43) Conspire to control price of cigarettes (price fixing)		(86) Support tobacco cultivation

### Sorting and rating of tobacco industry activities

Thirty-one participants took part in the sorting and rating of statements. For the sorting, each participant was asked to group the statements into groups “in a way that makes sense to you.”^10^^–^12 The only restrictions in this sorting task are that there cannot be: (a) a pile consisting of one item; (b) a pile consisting of all items; or (c) a “miscellaneous” group (any item thought to be unique was to be put in its own pile). Each participant was then asked to give a brief label for each pile that summarised the concept contained in their piles or groupings of cards. The participants were then asked to rate the 86 statements with these instructions: “Rate each statements on a 1 to 5 scale for its relative importance (compared to the rest of the statements) using 1 if the statement is relatively unimportant for tobacco control and 5 if the statement is extremely important.” Because participants are unlikely to brainstorm statements that are totally unimportant with respect to the focus, it was stressed that the rating should be considered a relative judgment of the importance of each item to all the other items brainstormed.

In addition, the participants also rated the statements for the potential feasibility of collecting or obtaining the data related to the statement. Again this was rated on a 1–5 scale with 1 being among the least feasible to collect and 5 being among the most feasible.

### Data analysis

The analysis begins with construction from the sort information of a matrix of similarities that shows the number of sorters who placed any two statements in the same pile regardless of what else was sorted with them.^10^ This matrix is the input for a non-metric multidimensional scaling (MDS) analysis with a two dimensional solution.^13^ ^14^ The analysis yields a two dimensional (x,y) configuration of the set of statements based on the criterion that statements piled together most often are located more proximately in two dimensional space while those piled together less frequently are further apart. The MDS configuration of the statement points is graphed in two dimensions.

The x,y configuration is the input for the hierarchical cluster analysis utilising Ward’s algorithm as the basis for defining a cluster.^15^ Using the MDS configuration as input to the cluster analysis in effect forces the cluster analysis to partition the MDS configuration into non-overlapping clusters in two dimensional space. There is no simple mathematical criterion by which a final number of clusters can be selected. The procedure that was followed to examine an initial cluster solution was the maximum desirable for interpretation in this context. Then, successively lower cluster solutions were examined, with a judgment made at each level about whether the merger seemed substantively reasonable. The pattern of judgments of the suitability of different cluster solutions was examined and the final number of clusters selected to preserve the most detail and still yield substantively interpretable clusters of statements.

### Generation of map

**Figure 1:**
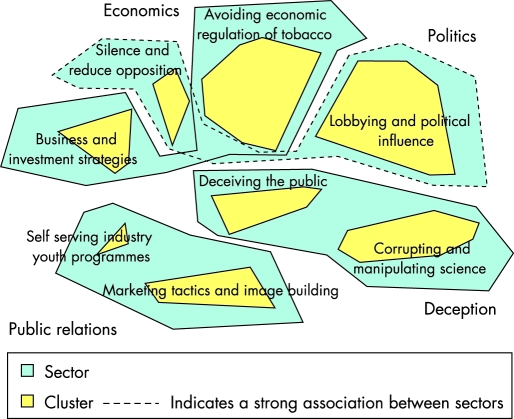
Concept map of industry activities to block tobacco control in South East Asia.

The “point map” (not shown) displays the location of all the brainstormed statements as determined by MDS, with statements closer to each other generally expected to be more similar in meaning. A “cluster map” is also generated that displays the original statement points enclosed by polygon-shaped boundaries that indicate the clusters. Both maps form the foundation of the final map, which can be seen in figure 1. Cluster labels were generated through face to face discussion and interpretation with participants.

### Rating importance and feasibility

The 1-to-5 importance and feasibility rating data are averaged across individuals for each item and each cluster. This rating information is depicted graphically in a “point rating map” showing the original point map with the average rating per item displayed as vertical columns in the third dimension, and in a “cluster rating map” that shows the cluster average rating using the third dimension.

### Pattern match of importance and feasibility

Pattern matching is both a statistical and graphic analysis.^16^ ^17^ Graphically, a pattern match is portrayed using a “ladder graph” that consists of two vertical axes (one for each “pattern,” in this case levels of importance and feasibility). The figure is called a “ladder” graph because strong agreement between the patterns will result in a set of near horizontal lines that look like a ladder. In this case, the patterns refer to the importance and feasibility ratings. The vertical axes display the rank order of cluster mean scores. The horizontal lines joining the two vertical axes display the correlation between the clusters using a Pearson product moment. The pattern match enables immediate identification of which cluster areas show the greatest consensus or discordance.

### Go zones

The “go zone” is a bivariate plot displaying both the relative importance and feasibility ratings of each statement in the cluster. Each cluster has a “go zone.” The “go zone” provides a detailed description of the relative importance and feasibility of the specific industry tactics. It, therefore, can be used to identify immediately and visually the statements that were judged by participants to be simultaneously above average in importance and feasibility—that is, optimal areas for action planning.

## RESULTS

At the regional workshop in Bangkok, the participants reviewed and interpreted the results, and came to consensus on final labels to describe each cluster and sector of the map. The final labelled concept map is shown in figure 1. The depicted map resulted in eight clusters of statements reflecting activities the industry used to block tobacco control. The clusters were labelled by the participants as (clockwise to centre): “Avoiding economic regulation of tobacco”; “Lobbying and political influence”; “Corrupting and manipulating science”; “Marketing tactics and image building”; “Self serving industry youth programme”; “Business and investment strategies”; “Silence and reduce opposition”; and “Deceiving the public.” Of the eight clusters, the participants identified four main sectors for the map, which were labelled: “Economics,” “Politics,” “Deception” and “Public relations.”

In figure 1, the sectors “Economics” and “Politics” were intertwined, which indicates a strong association since points located near each other on the map represent stronger associations and connectedness and points further away indicate more distant associations.

The cluster, “Self serving industry youth programmes” was closely connected to both “Business and investment strategies” as well as “Marketing tactics and image building”.

Equidistant to all clusters is “Deceiving the public” which, for participants, seemed to represent a central tactic of the tobacco industry in South East Asian countries.

The average importance and feasibility rating for each cluster is given in table 2.

**Table 2:** Importance and feasibility ratings for each concept map cluster (1–5 scale)

Cluster		Importance	Feasibility
		Mean rating (SD)	Mean rating (SD)
Marketing tactics and image building		4.04 (0.91)	3.71 (1.18)
Avoiding economic regulation on tobacco		3.93 (0.92)	3.00 (1.15)
Lobbying and political influence		3.83 (1.03)	2.54 (1.07)
Silence and reduce opposition		3.69 (1.09)	2.25 (1.04)
Deceiving the public		3.65 (0.97)	2.80 (1.10)
Self serving industry youth programme		3.63 (0.99)	3.51 (1.08)
Business and investment strategies		3.41 (1.13)	2.87 (1.18)
Corrupting and manipulating science		3.39 (1.10)	2.69 (1.06)

Of all clusters “Marketing tactics and image building” had the highest ratings for both importance and feasibility. Table 3 lists all the individual statements for both the importance and feasibility ratings for this cluster (other cluster ratings and importance are not shown). Developing display and promotional materials (No 83) at point of purchase was a tactic used by the industry that was rated high in both importance and feasibility for tracking and surveillance.

**Table 3:** Importance and feasibility ratings for marketing tactics and image building cluster

Statements		No	Importance	No	Feasibility
			Mean rating (SD)		Mean rating (SD)
(49) Promote tobacco products through use of young, pretty girls		30	4.30 (0.92)	26	4.04 (1.11)
(58) Circumvent ad bans through indirect promotions and sponsorships		31	4.23 (0.76)	26	3.46 (1.21)
(79) Target women through the use of western images of female empowerment		31	4.23 (0.84)	26	3.65 (1.29)
(60) Sponsor sports and music concerts		31	4.19 (0.79)	26	3.92 (1.26)
(83) Develop display and promotional materials at point of purchase		31	4.19 (0.65)	26	4.23 (1.11)
(59) Circumvent ad bans through brand stretching		31	4.13 (0.96)	26	3.88 (1.31)
(84) Promote tobacco through free giveaways		31	4.06 (0.89)	26	3.69 (1.01)
(65) Engage in free sampling of tobacco products		31	3.94 (1.00)	26	3.65 (1.13)
(73) Publicise corporate and social responsibility activities to enhance public image		31	3.87 (0.96)	26	3.69 (1.12)
(51) Target poor by images associating tobacco with success		31	3.87 (1.20)	26	3.62 (1.27)
(7) Evade advertising bans through trans-border broadcasting		30	3.87 (0.82)	26	3.36 (1.11)
(13) Publicise philanthropy contributions		31	3.84 (1.04)	26	3.77 (1.11)
(69) Highlight philanthropic contributions (for example, medical missions and disaster relief)		30	3.83 (0.99)	26	3.31 (1.29)

**Figure 2:**
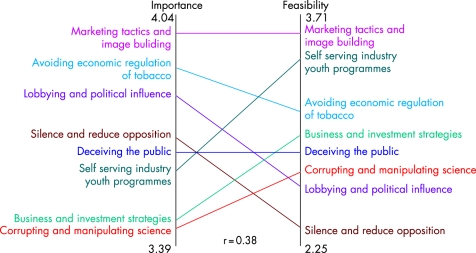
Pattern match for importance and feasibility of industry activity clusters (correlation coefficient, r). Relative importance: 1 = relatively unimportant (compared with the rest of the statements); 2 = somewhat important; 3 = moderately important; 4 = very important; 5 = extremely important (compared with the rest of the statements). Relative feasibility: 1 =  not at all feasible; 2 = not very feasible; 3 = somewhat feasible; 4 = moderately feasible; 5 = very feasible.

Figure 2 illustrates a pattern match that describes the degree of agreement between the ratings of importance and feasibility. The results of this match show that there are some discrepancies between the ratings of importance and feasibility with an overall correlation of 0.38. The pattern match shows, for example, that, on average, the “Marketing tactics and image building” cluster was judged both most important and most feasible for tobacco control to address. For some clusters a relatively important industry strategy might be associated with a feasibility of collecting data on that cluster that is relatively lower (for example, “Lobbying and political influence”) while for others the reverse may be true (for example, “Self serving industry youth programmes”).

### Go zone analysis

Because of the relatively high importance and feasibility of the statements in the Marketing Tactics and Image Building cluster, the individual statements were examined with a go zone analysis. The “go zone” identified statements with the highest importance and feasibility ratings in this cluster. In this case, the statements that were above the cluster average for both importance and feasibility were 49, 59, 60 and 83 (see table 3).

## DISCUSSION

The twin purposes of this study—generating consensus for monitoring and surveillance and building capacity to improve tobacco control planning—called for an approach that was both objective and informed by expertise. The concept mapping methodology used here was a rigorous alternative to expert panel and focus group techniques often employed for such purposes. Nevertheless, the method does have weaknesses that should be noted and that suggest lines of development for future research. As in any research that does not use random sampling, it is possible that these results are specific to the expert panel sampled and not generaliseable to other experts or practitioner populations that might have been included. Thus, subsequent research that replicates this study is warranted. In addition, future studies could also focus on inter-country differences, which would provide valuable insights into country specific tobacco industry strategies.

In addition, this study is, like all small sample methods, subject to the vagaries of low sample sizes, and the resultant limits this places on statistical estimates. While these are important limitations, they are no more salient in this case than in other small sample methods. These limitations could be addressed in subsequent research by replicating this study with different samples of participants and either different variations of this methodology or with alternative methods. Indeed, the striking similarity of these results with those of an independently conducted study in the United States that used the same methodology make it less likely that these results are spurious or the result of chance. Additional independent replications would help delineate the degree to which these results are generalisable.

### Use in generating consensus

Over the past five years, since the inception of the Rockefeller funded Trading Tobacco for Heath (TTFH) initiative, the South East Asia Tobacco Control Alliance (SEATCA) has been working with government partners, civil society agencies and tobacco control advocates to develop country specific tobacco control agendas with a regional perspective and to expand tobacco control partnerships and networks locally and globally. Concept mapping was used as a method for identifying a shared perspective and a common vision in a region where there is considerable variation across tobacco industry activities, government infrastructure and socioeconomic development. Countries that participated had both similarities and differences. The starting points in terms of experience in tobacco control and resources available differed dramatically, with countries like Thailand with 30 years of experience compared to Cambodia with less than a decade of tobacco control efforts. Despite these differences, the project needed to involve experts and stakeholder agencies in the region from diverse backgrounds to make use of their knowledge and expertise.

The FCTC requires that countries collect data on implementation of tobacco control measures and monitor compliance of tobacco control policies for consistency with FCTC. Such a surveillance system needs also to be country specific to inform local policies and programmes but also include data that are comparable across countries to inform regional efforts. Participants agreed upon the importance for each country to have their own local data balanced with the need for standardised data across the region. The use of concept mapping in this project illustrates a process and framework for identifying common data needs and measurement tools. Concept mapping is both a participatory and statistical methodology that allowed people from four different countries, with different areas and levels of expertise to identify common ground and to come to consensus on which tobacco industry strategies were most important and feasible to track. This project represented a participatory effort to identify the important areas for tracking industry strategies and for building consensus across the four countries.

### Use in measurement development

Figure 1 could be used as the basis for the development of an index of tobacco tactics. To do so would require that each of the clusters be operationalised. The statements within each cluster suggest potential elements that might be measured as a part of that index. For instance, one statement in the cluster “Marketing tactics and image building” was “Develop display and promotional materials at point of purchase.” This could be operationalised at the individual country level by developing a survey tool that would measure the level of point of purchase advertising at point of sale, through various constructs such as number of posters on doors, number of display cases, etc. Indeed, the project developed a point of purchase tracking tool to help local researchers track and monitor the level of point of purchase advertising.

### Use in tobacco control planning

The concept mapping process, especially the priority ratings of importance and feasibility, can provide a high level strategic view of industry tactics and areas of priority for tobacco control monitoring and surveillance. The project gathered information concerning the perceived feasibility of collecting reliable and valid data on specific tobacco industry strategies. As shown in the pattern match (fig 2) there were several clusters of industry activities that were deemed important. However, in low resource settings such as South East Asia, it is important to focus on areas where there was consensus on both feasibility and importance. The strategies identified by the experts who participated in the concept mapping process are substantiated by the literature. Saloojee and Dagli describe various methods used by the tobacco industry to counter tobacco control efforts and discuss these tactics within the context of the ongoing globalisation of tobacco use.^18^ Saloojee and Dagli identify nine focal points of the industry’s efforts: engineering consent, mobilising corporate resources, manufacturing doubt, protecting corporate rights, gathering intelligence, controlling the agenda, peddling influence, promoting voluntary codes and pre-emptive legislation, and opening markets through trade sanctions and corruption. However, such reviews were not conducted to assist with the creation of a metric of tobacco industry tactics for surveillance. And while many tactics are important, ratings on feasibility show that tactics such as lobbying and political influence are harder areas on which to collect reliable and valid data.

This study builds on previous work in the United States using an identical methodology, and the results are strikingly similar.^3^ Table 4 shows the clusters for the map reported here and the comparable clusters from the US study. The only cluster in this study that is not represented at the cluster level in the US map was the cluster “Self serving industry youth programmes.” Although there were statements in the US map that pertained to youth, they did not form a discrete cluster. This suggests that the tobacco industry may have a more salient youth effort at this point in South East Asia than they do in the United States, where significant efforts have long been under way to undermine the industry’s efforts. All of the US clusters have at least some counterpart in the South East Asia map. The pattern match in this study suggests some of the major measurement and surveillance challenges. While marketing and youth programme tactics are relatively feasible to measure, the other clusters present more difficulties. Two of the top four clusters in importance—“lobbying and political influence” and “silence and reduce opposition”—are the least feasible areas for measurement.

**Table 4:** Comparison of clusters in South East Asia map and US map

South East Asia map		US map
Avoiding economic regulation on tobacco		Legal and economic intimidation
Lobbying and political influence		Lobbying and legislative strategy
		Usurping the agenda
Corrupting and manipulating science		Undermining science
Marketing tactics and image building		Public relations
		Usurping the agenda
Self serving industry youth programmes		
Business and investment strategies		Legal and economic intimidation
Deceiving the public		Media manipulation
		Creating the illusion of support
Silence and reduce opposition		Harassment

The concept map represents an empirically derived consensus of a panel of tobacco control experts and was intended as a guide for subsequent development of instruments to assess tobacco control strategies. The ratings provide further indication of areas where to focus—areas of both importance and feasibility (go zones). Of all strategies, point of purchase advertising (POP) had the highest mean scores in both importance and feasibility. In the wake of this study, for each of the four countries, detailed measurement and tracking tools were developed along with a shared protocol and database for monitoring and assessment of POP marketing.

This project has furthered our understanding of what strategies could be the focus of a surveillance effort. As countries begin to implement the FCTC and will need to conduct and report on tobacco control surveillance, lessons learned from this project will be instructive for how to develop a process that will be inclusive of the needs of many stakeholders and stakeholder groups both locally and internationally as well as highlight the possible items to include in a regional surveillance and tracking system that are important and feasible.

**What this paper adds**

- The Framework Convention for Tobacco Control (FCTC) requires that countries collect data on implementation of tobacco control measures and monitor compliance of tobacco control policies for consistency with FCTC. Such a surveillance system needs to be country specific to inform local policies and programmes but also must include data that are comparable across countries to inform regional efforts.
- This article builds upon previous work that was conducted in the United States using the same methodology.^19^
- Lessons learned from the model presented in this paper will be instructive for how to develop a process that will be inclusive of the needs of many stakeholders and stakeholder groups both locally and internationally.
- The paper also highlights possible items to include in a regional surveillance and tracking system that are both important and feasible in low income resource settings.
- Equally important, the paper identifies areas of measurement and surveillance challenges, such as tracking “lobbying and political influence.”
- Most importantly, the paper demonstrates the importance of understanding tobacco industry tactics to serve as a basis of what strategies could be the focus of a tobacco control surveillance effort.
